# Exploring the role of common regulatory variants in the etiology of late-onset Alzheimer’s disease

**DOI:** 10.1186/1750-1326-7-S1-S22

**Published:** 2012-02-07

**Authors:** Hao Wang, Min Zhu, Jian Wang, Yue Sun, Yu Tao, Qin He, Xiang Xu, Li Chen, David Saffen

**Affiliations:** 1 Institutes of Brain Science, State Key Laboratory for Medical Neurobiology, School of Life Science, Fudan University, China

## Background

Late-onset Alzheimer’s disease (LOAD) is a complex disorder with a significant genetic component (heritability ~ 0.8). It is distressingly common: in the United States, approximately 13% of individuals over the age of 65 and 43% of individuals over the age of 85 are estimated to have AD [2011 Alzheimer’s Disease Facts and Figures, Alzheimer’s Association]. Although specific mutations have been linked to familial forms of AD, mutations in AD candidate genes among LOAD cases are rare. For this reason there is currently great interest in identifying common genetic variants that contribute to this disorder. The goal of the current study is to quantify allele-specific differences in the expression of mRNAs encoding candidate LOAD susceptibility genes in human brain and elucidate the genetic basis for this differential expression. Haplotypes or combinations of genotypes that correlate with mRNA should be useful as markers in future genetic association studies aimed at identifying genes that contribute to AD risk and protection.

## Methods

We examined the expression of mRNAs for candidate LOAD genes in human brain using a novel PCR/second-generation DNA sequencing-based assay for quantifying allelic expression imbalance (AEI) of mRNA expression. Population distributions of log_2_AEI ratios were analyzed using a custom mathematical model that yields information concerning the number, locations, and relative contributions of *cis*-acting regulatory variants that influence mRNA expression.

## Results

We determined population distributions of log_2_AEI ratios for 24 candidate LOAD genes, including *ABCA1*, *ADAM17*, *AGER*, *BACE1*, *BACE2*, *BDNF*, *CALHM1*, *CH25H*, *CLU*, *GAB2*, *GSK3B*, *LRP1*, *MAPT*, *NTRK2*, *P25*, *PICALM*, *PION*, *PSEN1*, *PSEN2*, *SORCS1*, *SORL*, *TNFRSF1*, *TNFRS21 AND TNK1.* Among these, 22 genes showed frequent (> 0.1) and significant (> 1.2-fold) allele-specific differences in mRNA expression. Mathematical modeling predicted that individual genes are regulated by one to three *cis*-acting elements. We are currently working on identifying haplotypes or combinations of genotypes that predict high- or low-levels of mRNA expression for use as markers in genetic association studies. Combining this information with knowledge of the functions of genes within biological pathways implicated in LOAD, will allow predictions of the contributions of specific haplotypes to the disorder. For example, high-expression haplotypes for genes encoding proteins that increase the production of toxic A-beta, are predicted to increase risk of LOAD, while high-expression haplotypes for genes encoding proteins that increase the degradation or clearance of A-beta, are predicted in decrease the risk of LOAD. In addition to simply examining differences in haplotypes frequencies between case and control populations, the ability to classify haplotypes as risk-conferring or protective allows the calculation of polygenic risk scores to each individual in the case and control groups. If our classification scheme has validity, we would expect the mean polygenic risk scores for cases to be higher than controls. Association studies to test these ideas are currently in progress.

## Conclusions

We have successfully used a novel high-throughput assay for quantifying common variation in mRNA expression for LOAD candidate genes in human brain and are employing a custom mathematical model to infer the number, contribution and locations of *cis*-acting regulatory variants that influence mRNA expression. We plan to uses data within a polygenic model for LOAD risk to test the hypothesis that common regulatory variants contribute to the etiology of this disorder.

